# Crosstalk Regulation Between Bacterial Chromosome Replication and Chromosome Partitioning

**DOI:** 10.3389/fmicb.2019.00279

**Published:** 2019-02-26

**Authors:** Gregory T. Marczynski, Kenny Petit, Priya Patel

**Affiliations:** Department of Microbiology and Immunology, McGill University, Montreal, QC, Canada

**Keywords:** DnaA, GapR, PopZ, chromosome replication, partitioning, cell cycle

## Abstract

Despite much effort, the bacterial cell cycle has proved difficult to study and understand. Bacteria do not conform to the standard eukaryotic model of sequential cell-cycle phases. Instead, for example, bacteria overlap their phases of chromosome replication and chromosome partitioning. In “eukaryotic terms,” bacteria simultaneously perform “S-phase” and “mitosis” whose coordination is absolutely required for rapid growth and survival. In this review, we focus on the signaling “crosstalk,” meaning the signaling mechanisms that advantageously commit bacteria to start both chromosome replication and chromosome partitioning. After briefly reviewing the molecular mechanisms of replication and partitioning, we highlight the crosstalk research from *Bacillus subtilis*, *Vibrio cholerae,* and *Caulobacter crescentus*. As the initiator of chromosome replication, DnaA also mediates crosstalk in each of these model bacteria but not always in the same way. We next focus on the *C. crescentus* cell cycle and describe how it is revealing novel crosstalk mechanisms. Recent experiments show that the novel nucleoid associated protein GapR has a special role(s) in starting and separating the replicating chromosomes, so that upon asymmetric cell division, the new chromosomes acquire different fates in *C. crescentus*’s distinct replicating and non-replicating cell types. The *C. crescentus* PopZ protein forms a special cell-pole organizing matrix that anchors the chromosomes through their centromere-like DNA sequences near the origin of replication. We also describe how PopZ anchors and interacts with several key cell-cycle regulators, thereby providing an organized subcellular environment for more novel crosstalk mechanisms.

## Introduction: Bacterial Cell Cycles Require Crosstalk and Coordination

To ensure their survival and proliferation, bacteria overlap and compress cell-cycle processes that are complex and time consuming. This overlap in bacteria contrasts with eukaryotes, which have sequential and non-overlapping phases for chromosome replication (S-phase), partition/segregation (mitosis), and cell-division/cytokinesis. Each eukaryotic phase of the cell cycle takes time, and while their sequential ordering enables accurate checkpoint controls, this system also prolongs the cell cycle and consequently limits the growth rates. Bacteria overcome this limitation and increase their growth rates by overlapping the phases of chromosome replication, partition/segregation, and cell wall growth/cell division (Helmstetter et al., 1968). The initiation of chromosome replication immediately precedes the initiation of chromosome partitioning and chromosome movement into separate cell spaces that will eventually become the daughter cells at cell division (Toro and Shapiro, 2010). This close temporal link suggests that it would be especially advantageous to co-regulate replication and partitioning. In a previous review article from our lab, we argued that eubacteria use one origin of replication (*ori*) per chromosome not because they are simpler organisms, but because a single *ori* allows for a more rapid and efficient control of replication (Marczynski et al., 2015). Bacterial chromosomes with one *ori* can more easily respond to many inputs both from outside and from inside the cell. We will argue that input/signals from inside the cell and crosstalk/signals with chromosome partitioning (*par*) systems are especially important. While most studies illustrate *par* components signaling replication, we will also highlight recent studies of crosstalk in the reverse direction. However, before presenting concrete examples of crosstalk, we will first outline the basic features of both *ori* and *par* systems and emphasize their potential for regulation.

## Origins of Replication Receive Signals and Dynamic Protein Assemblies

Like transcription promoters, bacterial *oris* are platforms for assembling replication proteins and their regulators (Kornberg and Baker, 1992). The *Escherichia coli oriC* and DnaA model for initiating chromosome replication has revealed the most detailed molecular mechanisms that operate inside *oris* (Kaguni, 2011; Skarstad and Katayama, 2013; Kaur et al., 2014). In broad outline, a bacterial *ori* is a specific place where the DnaA protein binds an array of DnaA boxes to self-assemble and then to promote the assembly of the downstream replication proteins (Wolanski et al., 2014a,b).

In *E. coli*, chromosome replication starts from one “*oriC*” when a threshold level of activated DnaA (ATP bound ATP-DnaA) is reached (Katayama et al., 2010; Skarstad and Katayama, 2013). Both forms of DnaA, ATP-DnaA and ADP-DnaA, bind to the strong/high affinity DnaA boxes in *oriC*, but only the activated ATP-DnaA proteins will bind to weak DnaA box motifs and oligomerize on *oriC* through neighboring AAA+ domains (McGarry et al., 2004; Kawakami et al., 2005; Erzberger and Berger, 2006; Grimwade et al., 2018). Such DnaA self-assembly starts from strategically placed “anchor” DnaA boxes (Rozgaja et al., 2011), and the resulting protein-DNA structure (and possibly a helix) causes DNA unwinding and a further altered structure with new protein surfaces that recruit downstream replication proteins. More specifically, *oriC* DNA unwinding allows DnaA to recruit DnaB (the replicative DNA helicase) bound to DnaC, the helicase escort/loader, on to the single-stranded DNA of the AT-rich region (Mott and Berger, 2007). It is likely that two types of DnaA protein-DNA structures form on *oriC*; one that unwinds and keeps the AT-rich region open and single stranded and another DnaA-DNA structure that recruits and loads two DnaB hexamers around the single-stranded DNA. Once loaded, the two DnaB hexamers move apart, expanding the single-stranded DNA region, thereby permitting the recruitment of primase DnaG. Next, the DNA polymerase III holoenzyme composed of the Pol III and the beta-clamp (DnaN) is recruited, and together with the clamp-loading proteins, these form the “replisome” that synthesizes the complementary DNA strands (Kaguni, 2011; Skarstad and Katayama, 2013; Katayama, 2017).

Since most eubacteria use the DnaA protein to initiate chromosome replication (Wolanski et al., 2014a,b), DnaA and the assembly reactions at *oriC* are major targets for the regulators of chromosome replication (Wolanski et al., 2014a,b). Recent reviews have described many proposed and established regulators of replication, and an especially good review with fine graphic summaries was provided by Katayama et al. (2010).

Most importantly for our topic, DnaA assembly at *oriC* is dynamic, and *in vivo* there is probably both back and forth assembly and dis-assembly of DnaA until the critical amount of DnaA oligomerization and active structure formation is reached (Leonard and Grimwade, 2011; Kaur et al., 2014). This dynamic feature of *E. coli* replication initiation implies that there are many ways to shift the assembly versus dis-assembly of DnaA and DnaB. This process has the potential to integrate many signals that can be constantly added or subtracted in real time before the final commitment to replication is made. We will describe below how this view of dynamic *oriC*/DnaA assemblies helps us to understand the regulatory crosstalk with chromosome partitioning.

## DNA Partitioning Systems

Many bacteria use systems often called “*parABS”* for mitotic-like chromosome separation and partitioning into cell compartments, and their proximity to origins of replication (*oris*) suggests functional linkages (Livny et al., 2007). These partitioning systems were originally studied on large low-copy plasmids, and they account for faithful and consistent plasmid distribution to both progeny cells (Austin and Abeles, 1983; Ogura and Hiraga, 1983; Gerdes et al., 1985). Despite much effort, exactly how the *parABS* systems work to move and to position plasmid and chromosome DNAs remains incompletely understood and in parts controversial (Gerdes et al., 2010). Here we want to present the basic information and sketch what appear to us the most relevant models for our topic. Knowledge of the detailed mechanisms is required not just to understand how *parABS* systems work to partition DNA but also to understand and speculate how evolution has harnessed these systems for other functions and particularly for crosstalk with chromosome replication. With respect to deep evolutionary potentials, *parABS* systems have also been harnessed for protein positioning and localization, as, for example, organizing chemotaxis proteins and other large protein assemblies (Vecchiarelli et al., 2012).

In bare outline, the three-component *parABS* system works as follows: The *parS* DNA acts as a “centromere-like” locus with specific DNA sequences that bind and hold ParB proteins. ParA protein binds and hydrolyses ATP, and it somehow imparts motion to the ParB-*parS* complex through interactions with ParB. These basic functions need to be controlled and organized by regulators and structures that change during the cell cycle. As a main topic, we will address some key regulators and structures below, including, for example, the cell-pole proteins that anchor the chromosome ParB-*parS* complexes.

There are several significant variations to the above bare outline of *parABS* system. For example, some bacteria apparently use several *parS* loci, while others appear to use just one. *Bacillus subtilis* probably uses 10 *parS* loci and 8/10 loci cluster toward the *oriC* side of the chromosome (Breier and Grossman, 2007). *Myxococcus xanthus* may use as many as 22 *parS* loci, likewise near its *oriC*, for partitioning its exceptionally large 9.1 Mb chromosome (Iniesta, 2014). In contrast, the *Caulobacter crescentus* (Mohl and Gober, 1997) and the *Vibrio cholerae* (Espinosa et al., 2017) chromosomes appear to use just one *parS* per chromosome, and these single *parS* loci are likewise closely linked to their corresponding *oriCs*. Why does one bacterium need one *parS* and another several? There is no good answer yet, but this distinction may be too simplistic. For example, a recent study showed that *C. crescentus* has several yet substantially weaker ParB-binding sites (Tran et al., 2018), and it may be more correct to speak of a “*parS* region” surrounding the origin of replication as described further below.

There are also significant variations in how ParB binds DNA to create a “centromere-like” locus. ParB binds specifically to *parS* DNA and less specifically to other parts of the chromosome. First, ParB binds specifically to an inverted DNA repeat that is typical of many standard dimeric helix-turn-helix DNA-binding proteins, and these sites are easily found and used to identify *parS* sites in most bacterial genomes (Livny et al., 2007; Iniesta, 2014). However, ParB is reported to have additional modes of DNA binding. *In vivo* cross-linking and transcription reporter experiments imply that ParB binds to *parS* sites and then spreads to adjacent DNA as if forming a filament across the DNA to distant sites. It is not likely that “spreading” is an experimental artifact because spreading is required for partitioning. ParB mutants that do not spread do not partition DNA (Rodionov et al., 1999; Graham et al., 2014).

The exact DNA/protein structure(s) of these “spreading” ParB molecules is not known, but interactions can be inferred from crystal structures (Chen et al., 2015). ParB can bind other ParB molecules through lateral contacts that reach adjacent DNA and through bridging contacts that bring distant DNAs together with loops. This capacity for non-specific DNA binding suggests that ParB can be classified as one among many nucleoid-associated proteins (NAPs) that compact and organize bacterial chromosomes. Recently, a ParB “caging model” has been proposed whereby *parS* organizes a large chromosome subdomain through dynamic ParB-ParB and ParB-DNA interactions (Funnell, 2016). This model is further supported by *in vitro* experiments with magnetic tweezers, which suggest that the overall ParB-DNA complex is not well ordered and vaguely resembles a phase separation from the rest of the nucleoid (Taylor et al., 2015). In summary, considering the proximity of *parS* to *oriC*, ParB protein certainly has the potential to influence chromosome replication, and we will describe specific examples below.

## Partition Protein Para can be a Motor and a Regulator

The preceding observations argue that ParA imparts motion not just to a small ParB-*parS* locus but also to a large ParB-DNA subdomain of the chromosome. Exactly how ParA drives ParB-DNA motion also remains controversial. However, ParA has several established and speculative properties that enable it to serve both as a propeller and as a regulator. We will focus below on two properties required for regulation: First, we explain that ParA can act like a “molecular switch” and second, we explain that ParA (like ParB) can bind and influence large domains of DNA.

ParA “switches” within a biochemical cycle: ParA monomers bind ATP, the ParA-ATP dimerizes, and this form binds DNA non-specifically. ATP hydrolysis creates ParA-ADP molecules, which disassociate from the DNA as monomers. When ParA binds ParB, specific protein-protein contacts stimulate ATP hydrolysis, thereby resetting the ParA-ATP/DNA binding versus ParA-ADP/DNA release cycle (Vecchiarelli et al., 2010). A protein contact switch seems ideal for regulation, and as an interesting example, we will describe below how *Bacillus subtilis* has harnessed ParA to also regulate chromosome replication through direct contacts with DnaA.

Exactly how this ParA cycle drives ParB-DNA motion remains controversial. It is also not clear if propulsion and switching/regulation are separable functions. Here we can only superficially comment on this literature, and we will focus on how ParA binds to the nucleoid. For example, it has been proposed that ParA binds ParB and then retracts to pull the ParB-DNA along its path. This could be an active process where ParA imparts the force of motion or it could be a more passive mechanism, for example, a “catch and release” mechanism whereby ParA guides and biases a random “DNA flapping” motion. ParA may be organized as “microtubule-like” or as “cloud-like” structures that move forward and recede by assembly and dis-assembly. The literature is not consistent. However, there are credible reports that during partition, ParA forms dynamic cloud-like patterns on the surface of the nucleoid, and this pattern is interpreted as a gradient that recedes and seems to draw the ParB bound to *parS* (Hatano and Niki, 2010; Ah-Seng et al., 2013). Nucleoid patterning by ParA proteins resembles membrane patterning by the *E. coli* MinCDE system (Vecchiarelli et al., 2012), which imparts positional information for cell division, so that the septum forms at mid-cell (Lutkenhaus, 2007). Furthermore, the ParA and Min proteins belong to the same class of ATPases, and their mechanisms for molecular positioning may be fundamentally similar (Vecchiarelli et al., 2012).

Ietswaart et al. have presented an important synthesis between what seemed at first to be distinct and contradictory par mechanisms (Ietswaart et al., 2014). They demonstrate that the *par* system stimulates plasmid DNA motion above the random Brownian motion kinetics, thereby demonstrating that the *par* system can impart an active force and does not simply bias a random motion. Also, very importantly, Ietswaart et al. have argued that nucleoid structure plays an essential role in ordering the bound ParA-ATP structures. For example, helical nucleoid folds might provide grooves for channeling ParA-ATP aggregates into filaments or elongated clouds. Their model requires linear arrays of DNA-bound ParA-ATP and not necessarily that they be microtubule-like filaments. In other words, ParA-ATP linearity imparts the directionality to DNA motion and short disjoint filaments or elongated clouds (where individual ParA-ATP dimers bound to the nucleoid need not touch) will equally satisfy their model. In summary, the *par* literature argues that both ParA and ParB shape and respond to the structure of the nucleoid. Consequently, NAPs should significantly impact both chromosome replication and its partitioning. We will therefore discuss NAPs as regulators further below.

## Established Examples of Crosstalk: The *Bacillus Subtilis* System

*Bacillus subtilis* provides clear examples of crosstalk and a series of papers provide the best and earliest evidence. For example, early studies showed that *B. subtilis*, Spo0J(ParB), is required for the normal positioning of the *oriC* region and for restricting its replication. Wild type cells prior to replication place their *oriC* regions at the lateral mid-cell position and when they duplicate their *oriC* regions, they position them around the cell quarter-length positions. However, in *spo0J(parB)-*null strains, the duplicated *oriC* regions are positioned significantly closer together and toward the mid-cell. Interestingly, these *spo0J(parB)-*null strains had more *oriC* DNA per cell, as determined by flow cytometry. Apparently, *spo0J(parB)-*null cells had increased chromosome content from an excessive and/or an asynchronous initiation of DNA replication from *oriC* (Lee et al., 2003).

One general question is whether asynchronous firing of *B. subtilis oriC* was caused indirectly by *oriC* mislocalization or whether the ParAB system directly interacts with the replication system. Later studies showed that the *B. subtilis* ParAB proteins directly target DnaA (Murray and Errington, 2008). Using fluorescence-tagged ParA and ParB proteins, Murray and Errington showed that these proteins dynamically localize as specific foci (spots) near *B. subtilis* cell poles and nucleoids and that ParA can both inhibit and activate DnaA to alter chromosome replication. The inferred cytogenetic interactions between ParA and DnaA were supported by direct *in vivo* crosslinking and two-hybrid assays. In addition to this direct mechanistic link, this article also made several other interesting observations: For example, *parA*-null mutants behave like wild-type cells arguing for redundant or multiple regulatory inputs. Revealing the hidden cell-cycle interactions required assaying mutant protein forms. For example, revealing DnaA-dependent ParA foci at *oriC* required expressing a fluorescent ParA protein that bound ATP but did not bind DNA. Presumably, the weaker binding of ParA to DnaA protein at *oriC* would be otherwise obscured by its stronger binding to the larger/bulkier chromosome DNA. Similarly, revealing ParB-dependent ParA foci required fluorescent ParA that was deficient for ATPase and therefore apparently remained bound for longer times to the DNA.

Furthermore, the cell-cycle roles of ParAB were originally hidden because *parAB* mutants were first classified as sporulation genes. ParB was called Spo0J because null alleles were blocked in the earliest 0-stage of sporulation. ParA was called Soj, “suppressor of spo gene J,” because its null alleles allowed sporulation of spo0J null strains (Ireton et al., 1994; Quisel and Grossman, 2000). We now know that sporulation is inhibited by ParA (Soj), which requires ParA-ATP dimerization and that ParB (Spo0J) counteracts ParA (Soj) by stimulating ParA-ATP hydrolysis. Murray and Errington also showed that ParA (Soj) acts through the Sda-dependent DNA replication checkpoint (Murray and Errington, 2008). Sporulation is not just a simple response to starvation. Sporulation also requires passing several checkpoints and conditions that perturb chromosome replication block sporulation by expressing a sporulation inhibitor, Sda (Ruvolo et al., 2006). Most interestingly, the transcription promoter of *sda* has many DnaA boxes, and like *oriC*, it essentially acts as a sensor for DnaA activity. In other words, one had to look through one layer of regulation (Sda check point regulation) to see the other layer of *oriC*/DnaA regulation. Note also that both sporulation and chromosome replication are long processes that require a “full commitment” following a “deliberation process” with multiple inputs, and that evolution has recruited DnaA in both cases as an integrating component.

Subsequent studies showed how ParA changes DnaA oligomerization at the *B. subtilis oriC*. For example, Scholefield et al. showed that the initiation of chromosome replication is inhibited by monomeric ParA-ADP (Soj) and conversely activated by dimeric ParA-ATP (Scholefield et al., 2011). This study also identified specific amino-acid contacts on coregulator ParB (Spo0J) that touch ParA and “flip the switch” to its inactive form. Next, in their following paper, Scholefield et al. demonstrated specific amino-acid contacts between ParA and DnaA with both molecular-genetic and biochemical (e.g. SPR sensorgram and crosslinking) experiments. Most impressively, this study showed that monomeric ParA represses *oriC* replication by depolymerizing DnaA (Scholefield et al., 2012). These experiments used a functional double-cysteine version of DnaA (DnaA-CC) that allowed stable crosslinking of the DnaA-CC oligomers during *in vitro* and *in vivo* experiments. These oligomers presumably reflect the assembly of the DnaA-*oriC* DNA complexes, and their summary model implies that monomer ParA acts as a negative input during the dynamic assembly and dis-assembly process that tips *oriC* either toward or away from replication.

Recent microscopic studies have more directly confirmed this rapid assembly and dis-assembly model of DnaA at *B. subtilis oriC* and the proposed regulatory roles of ParA (Soj) in this dynamic process (Schenk et al., 2017). More specifically, “FRAP” fluorescence recovery and photobleaching analysis of a functional fluorescent YFP-DnaA protein showed that DnaA is bound to *oriC* with a short half-time of only 2.5 s. As predicted, a genetic deletion of *parA* (*soj*) increased the DnaA residence time at *oriC* and this in turn caused over-replication of the chromosome, presumably by shifting the equilibrium more frequently toward DnaA-*oriC* DNA complex formation. Furthermore, single-molecule YFP-DnaA microscopy showed that DnaA oscillates between polar-oriented *oriC* foci with a very short ~2 s periodicity. This last observation unexpectedly shows that DnaA can behave more like the *par* and *min* (cell division) system proteins than previously suspected (Schenk et al., 2017).

The overall view that emerges from these studies is that ParA (Soj) is an important *oriC*/DnaA regulator or more accurately, a key regulatory input. This regulation is not essential but instead seems to fine tune the cell cycle in growing cells and their timely exit into sporulation. ParA (Soj) can either delay or advance the start of *oriC* replication depending on its monomer versus dimer states and its contacts with ParB (Spo0J). However, exactly how these factors link *oriC*/DnaA regulation to chromosome movements and perhaps to other cell-cycle processes remains vague and speculative.

## Established Examples of Crosstalk: The *Vibrio Cholerae* System

*Vibrio cholerae* presents another interesting, evolutionary very divergent and well-studied system for addressing chromosome replication and partitioning. This topic has recently been well reviewed (Espinosa et al., 2017). *V. cholerae* is closely related to *E. coli*, and while these bacteria have expected similarities, they also have some very surprising differences. For example, the *V. cholerae oriC* and the *E. coli oriC* seem to function and use DnaA very similarly. However, unlike *E. coli*, *V. cholerae* has two chromosomes, one replicated by an *E. coli*-like *oriC* (Chrom I) and the other by a distinct plasmid-like origin of replication (Chrom II). The *V. cholerae* Chrom I and *E. coli oriCs* have identical DnaA box distributions, and as expected, DnaA is the primary initiator (Egan and Waldor, 2003). In contrast, the *V. cholerae* Chrom II *ori* has only one DnaA box, and it instead uses an “iteron” organization, i.e., a long array of binding sites for the initiator protein RctB (Gerding et al., 2015). Yet, despite such major differences both Chrom I and II are well integrated into the *V. cholerae* cell cycle, and their replication is strictly timed (Espinosa et al., 2017).

*V. cholerae* Chrom I and II have evolved separate and specific replication and partitioning crosstalk systems. For example, the control of chromosome replication through ParA and ParB, seen above in *B. subtilis*, also seems to apply to the large chromosome (Chrom I) of *V. cholerae* (Kadoya et al., 2011). Interestingly, each Chrom I and II has its own chromosome-specific *parABS* system. Accordingly, Chrom I has corresponding *parA1*, *parB1,* and *parS1* linked to its *E. coli*-like *oriC*. Prior to the start of replication, this *V. cholerae par/oriC* DNA region is positioned at the cell pole. Deletion of either *parA1* or *parS1* caused delocalization away from the cell pole. Deletion of *parB1* caused a similar delocalization as expected, plus an increased *oriC* copy number indicating that lack of ParB1 causes over-replication. Therefore, as in *B. subtilis*, ParB1 limits ParA1 activity, which then presumably targets *oriC* through DnaA. This view is supported, by, for example, double *parB1* and *parA1* deletions, which reduce and restore approximately normal levels of *oriC* replication presumably by eliminating the stimulus of ParA1-ATP dimers. Unfortunately, direct evidence for ParA1 and *V. cholerae* DnaA interactions is lacking. It is tempting to speculate that like *B. subtilis* ParA (Soj), the *V. cholerae* ParA1 also directly contacts the AAA+ domain of DnaA and more specifically that it too both stabilizes and destabilizes the DnaA structure on *oriC*. However, there are many ways to regulate DnaA activity, and considering the evolutionary distance between Gram (+) and Gram (−) bacteria, other mechanisms are likely, and the details of this broad outline need to be investigated.

The *V. cholerae* (*Vc*) Chrom II system is significantly different from Chrom I: Its *ori* is flanked by two genetic loci *rctA* and *rctB* (Egan and Waldor, 2003). While *rctB* simply encodes the DNA-binding initiator protein, the *rctA* locus seems to be a complex regulatory system with the *Vc parS2* “centromere” embedded among its regulatory elements (Gerding et al., 2015). Also, Chrom II seems to have an interesting parallel regulation with that of the *Caulobacter crescentus* (*Ccr*) chromosome, which will be described further below: As with most *parABS* systems, the *Vc parS* centromere locus in *rctA* binds *Vc* ParB2 and the *Ccr parS* binds *Cr* ParB. However, very interestingly, both centromere loci also bind their main replication initiator proteins, *Vc* RctB (Gerding et al., 2015) and *Ccr* DnaA, respectively (Mera et al., 2014). This is probably an example of convergent functional evolution, since *Vc* RctB and *Ccr* DnaA are otherwise unrelated.

Despite these two clear examples of crosstalk, the details of their mechanisms, as far as they are known, appear to be very different. The details of *Ccr parS* and *Ccr* DnaA interactions will be described further below in the context of cell-cycle control. Here we will note some mechanistic similarities and differences. For example, the *rctA/parS2* locus of *Vc* Chrom II binds RctB protein and seems to repress replication by titrating RctB away from the nearby origin of replication (Yamaichi et al., 2011). This is clearly different than *Ccr* DnaA protein that binds *parS* to apparently trigger DNA movement. Also, RctB has at least two separate DNA-binding domains (Yamaichi et al., 2011), one to bind *rctA* DNA and the other to bind the iteron motifs inside the adjacent Chrom II *ori*. In contrast, DnaA uses its single domain IV to bind DnaA boxes in both *parS* and *ori* DNA (Mera et al., 2014). Moreover, *rctA/parS2* seems to be a more complex locus. Its small ORF does not seem to encode a functional protein, and instead, it seems to function by providing an RNA molecule, or as a platform for transcription activity (perhaps to alter DNA topology), and as a platform for binding proteins, including ParB2 (at the main *parS2* sequences) and RctB. Both ParB2 and RctB can bind and simultaneously occupy *rctA* DNA in what appears to be adjacent binding zones (Yamaichi et al., 2011). ParB2 binding to *rctA* DNA counteracts *rctA* repression of replication, yet ParB2 protein does not seem to displace the bound RctB protein. This last observation argues that simple RctB protein titration away from the *ori* does not obviously explain how the *rctA* locus acts through RctB protein to repress replication or how ParB2 binding counteracts this effect. A fuller explanation is needed, and it may need to invoke altered protein and DNA structures.

Separate studies confirm the preceding antagonistic relationships among *rctA/parS2*, ParB2, and RctB, but the inferred mechanism does not involve RctB titration (Venkova-Canova et al., 2013). Instead, it was argued that RctB binds short 12-mer DNA sequences to activate replication and to longer 39-mer DNA sequences to repress replication. Apparently, ParB2 has two ways to relieve this repression. In the first way, ParB2 binds at *rctA/parS2* and spreads laterally across the DNA into a nearby 39-mer, thereby displacing RctB and relieving its repression. In the second way, ParB2 has a secondary intrinsic affinity for the 39-mer DNA, and so ParB2 competes for RctB repressor binding at a distant 39-mer without the spreading mechanism from *parS2*.

Furthermore, RctB and ParB2 provide a second level of crosstalk since they control transcription of the downstream *parAB2* operon. As observed in similar *par* systems, ParB2 binds *parS2/rctA* and auto-represses the *parAB* operon. However, RctB binding stimulates transcription, thereby increasing ParB2. Therefore, RctB and ParB2 have mutually antagonistic effects on both *parAB2* operon transaction and on Chrom II replication (Yamaichi et al., 2011; Gerding et al., 2015). Overall, these observations suggest a dynamic back and forth switch between *par* and *ori* control that is yet to be fully understood.

In summary, the *V. cholerae* two chromosome system provides interesting examples of *ori* and *par* crosstalk. At Chrom I, evolution has apparently conserved the ParB1, ParA1, and DnaA signaling pathway between *parS1* and the origin of replication. However, at Chrom II, evolution has modified the paralogous ParB2 protein to interact more directly with a very different type of origin of replication through direct contact or through competition with its iteron-binding protein RctB.

## The *Caulobacter Crescentus* Cell-Cycle Model for Crosstalk

**Figure 1:**
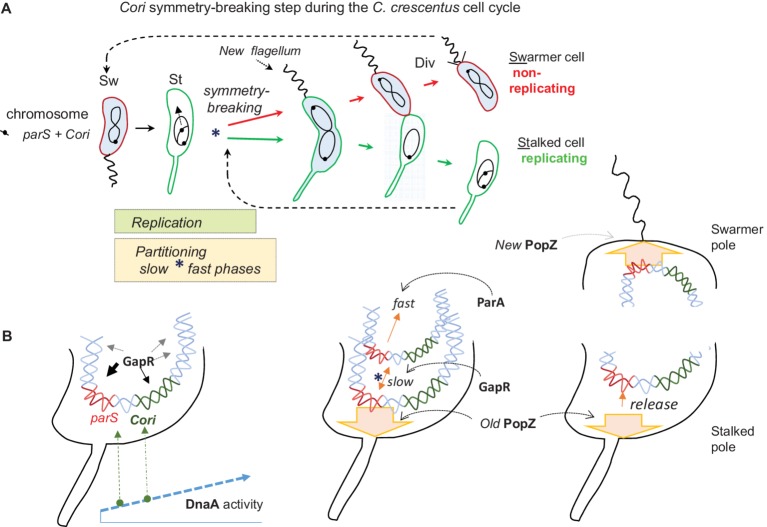
The *C. crescentus* cell cycle emphasizing asymmetric chromosome replication and partitioning. **(A)** The cell cycle conceptually starts on the left with the swarmer cell (Sw). It has one circular chromosome that is held at the flagellated cell pole by the centromere-like (*parS*) DNA linked to the origin of replication (*Cori*). The swarmer cell next differentiates into a non-motile and replicating stalked cell (St). Coincident with this cell differentiation, the chromosome replication and partitioning phases initiate apparently simultaneously, and they continue together for much of the cell cycle. The partitioning movement of *parS-Cori* has an initial slow phase that uses GapR protein and a later fast phase that requires the partitioning protein ParA (see text for further details). This slow partitioning phase overlaps the chromosome “symmetry breaking” step (*) of the cell cycle, which symmetrically channels the duplicated *parS-Cori* regions and eventually the entire chromosomes into distinct replicating (stalked cell) and non-replicating (swarmer cell) compartments. The blue cytoplasmic shading represents the activity (presence and phosphorylation) of the master cell-cycle regulator CtrA, which among many functions bind *Cori* to repress replication in swarmer cells. Asymmetric cell division (Div) proceeds with the return of CtrA activity and the building of a new polar flagellum. Eventually the two distinct cell types are formed. **(B)** A closer look at the cell poles during the above cell cycle. On the left, an early stalked cell pole where the *parS-Cori* region has been released from the PopZ matrix protein (not shown) and where rising DnaA activity first acts at *parS* DnaA boxes before acting at the *Cori* DnaA boxes. Although GapR binds broad regions of the chromosome, its strongest peaks are around the *parS-Cori* DNA. Next, a stalked cell pole immediately following the initiation of chromosome replication. One duplicated *parS-Cori* region reattaches to the old PopZ matrix at the stalked pole (symbolized by the broad arrow, the ParB bridge is not shown). The other duplicated *parS-Cori* region moves slowly away with the aid of GapR before its fast movement driven by ParA toward the other cell pole. On the right, both poles of a dividing cell. At the swarmer pole, the translocated *parS-Cori* region is attached to the new PopZ matrix that formed coincidentally with its arrival. At the opposite stalked pole, the *parS-Cori* region is released from PopZ roughly coincident with stalked cell reentry into another round of chromosome replication.

*Caulobacter crescentus* provides further evidence of *ori* and *par* crosstalk. As a chief advantage, this bacterium allows crosstalk studies in the context of a synchronized and well-studied cell cycle (Figure 1A). This is a “di-morphic” cell cycle where the transition from the “swarmer cell” to the “stalked cell” also marks the key steps of replication and chromosome partitioning. Conceptually, the cell cycle starts with the motile and non-replicating swarmer cell. Its chromosome replication is blocked by the CtrA regulator with five-binding sites inside the *C. crescentus* origin of replication (*Cori*) (Siam and Marczynski, 2000). The *C. crescentus parS* is only about 8 kb from *Cori* (Mohl and Gober, 1997), and this whole region of the chromosome is polarized and held near the flagellated cell pole by *parS-*binding ParB, which in turn is bound to a polar matrix protein called “PopZ” (Bowman et al., 2008). We will describe PopZ further below and argue that it can serve as a “hub” for many regulatory interactions, but the most conspicuous role for PopZ is to serve as the substrate that binds ParB, which thereby anchors the *parS* and *Cori* region in the swarmer cell (Bowman et al., 2008).

The cell-cycle transition from swarmer cell to stalked cell coincides with many molecular events that suggest *Cori* and *parS* crosstalk (Marczynski et al., 2015). While the swarmer cell ejects its flagellum and starts to grow its stalk (a tubular cell wall outgrowth), the CtrA protein is inactivated (dephosphorylated) and degraded, the *parS*-*Cori* region detaches from the cell pole, and the chromosome initiates replication from *Cori* (Toro and Shapiro, 2010). The initiation of chromosome partitioning is practically simultaneous with the initiation of chromosome replication, and both processes continue through most of the cell division cycle. Note especially that the dividing cell poles are different, one pole has a stalk, while the other is building a new flagellum, so this is an “asymmetric” cell division cycle (Figure 1). Eventually, one whole chromosome is placed in the nascent swarmer cell compartment, while the other chromosome is placed in the stalked cell compartment. In other words, with respect to chromosome replication, one chromosome will be placed into an inactive swarmer cell, and the other chromosome will be placed into an active stalked cell.

Such asymmetric cell division implies that the initiation of chromosome replication and partitioning coincide with the critical chromosome symmetry-splitting step of the cell cycle (Figure 1A). Time-lapse fluorescence microscopy showed that *parS-Cori* region DNA partitioning (visualized with fluorescent ParB) is a complex process involving at least the following steps: First *parS-Cori* separation, then *parS-Cori* discrimination, such that one *parS-Cori* region seems to be chosen for reattachment to the stalked pole. Then, the other (apparently unattached) *parS-Cori* region moves slowly away from the stalked pole to approximately the quarter-cell length position before moving more rapidly to the new swarmer pole (Shebelut et al., 2010). Further analysis showed that only the last fast-movement phase requires ParA ATPase activity and that the early slow movement of *parS-Cori* to the quarter-cell length position occurs faithfully when a dominant-negative ParA allele is expressed (Shebelut et al., 2010). Since fluorescently labeled ParB is bound to *parS* during this early slow-movement phase, then how does *parS*-ParB move without ParA?

More importantly, how do these early partitioning steps faithfully split chromosome symmetry to channel them toward two different cell fates (Figure 1A)? What are the regulators and the motors during the early partitioning steps? How do they communicate with chromosome replication? These questions are starting to be addressed in the following paragraphs.

## *C. Crescentus* DnaA also Signals Chromosome Partitioning

A study by Mera et al. implicated *C. crescentus* DnaA in chromosome partitioning (Mera et al., 2014). A conditional DnaA expression strain failed to initiate chromosome replication when DnaA was shut-off, as expected (Gorbatyuk and Marczynski, 2001), and as expected kept a single fluorescent ParB-*parS* centromere complex at the old stalked cell pole while the cell attempted to grow and divide. However, and very surprisingly, DnaA expression at low levels that could not initiate chromosome replication could still initiate and complete *parS-Cori* partitioning. Under these low DnaA conditions, many cells that had only a single, i.e. an un-replicated ParB-*parS* centromere complex could still move it completely from the old stalked pole to the new swarmer cell pole. Mera et al. clearly showed that DnaA binds the *parS* and that DnaA-ATP is required for this partitioning since a DnaA allele that does not bind ATP does not support partitioning. The view suggested by these results is that as DnaA activity rises (as both protein abundance and DnaA-ATP) during the swarmer cell to stalked cell transition, DnaA first acts at *parS* to perhaps commit the chromosome to partitioning before acting at *Cori* to commit it to chromosome replication (Figure 1B). This view is attractive considering that DnaA often acts as a global regulator of cell-cycle gene expression (Hottes et al., 2005) and chromosome replication (Gorbatyuk and Marczynski, 2001) and now apparently chromosome partitioning as well.

## Control by Nucleoid-Associated Proteins

Nucleoid organization can theoretically impact both chromosome replication and partitioning (Badrinarayanan et al., 2015). Unlike eukaryotic cells, bacteria do not possess histones, and instead, several small proteins called nucleoid-associated proteins (NAPs) compact and organize their genomes (Luijsterburg et al., 2006; Stavans and Oppenheim, 2006). Bacterial NAPs are not always conserved, but they share many features such as a small size, a high expression level, and a tight DNA binding (Krogh et al., 2018). NAPs impact DNA topology, which must be regulated for efficient transcription and replication (Donczew et al., 2014; Dorman and Dorman, 2016). For example, negatively supercoiled genes are more efficiently transcribed than positively supercoiled genes suggesting transcriptional control by NAPs (Sobetzko et al., 2012). The most investigated and one of the most conserved NAPs is the HU protein of *E. coli* (Ali Azam et al., 1999). HU exists as a homo- or hetero-dimer of *α* and β chains depending on the growth phase. DNA-binding affinity is different for each dimer, leading to differential nucleoid compaction and differential transcription between the growth phases. HU also stabilizes the pre-replication complex essential for the initiation of *E. coli oriC* replication (Chodavarapu et al., 2008). Other NAPs also influence the initiation of DNA replication. In *E. coli*, NAPs “FIS” and “IHF” repress and stimulate the initiation of DNA replication (Ryan et al., 2004; Wolanski et al., 2014a,b). In *B. subtilis*, the NAP “ROK” recruits and interacts with the bacterial replication initiator DnaA. ROK thereby directs DnaA to repress transcription and to help shape the nucleoid (Seid et al., 2017).

## *C. Crescentus* GapR is a Novel NAP that AIDS Chromosome Replication and Partitioning

In *C. crescentus*, the recently identified and now best characterized NAP “GapR” is implicated in cell-cycle control including chromosome replication and partitioning. GapR is an essential 89 amino-acid protein exclusively found in the alpha-proteobacteria, which are also known for their asymmetric cell division (Brilli et al., 2010). It is therefore tempting to speculate that GapR contributes to the chromosome asymmetry of *C. crescentus* (Figure 1A). Recent papers report that GapR has several relevant properties. For example, GapR binds DNA in AT-rich regulatory regions and next to highly expressed genes. Interestingly, the bulk distribution of GapR on the chromosome forms a gradient that decreases from the *parS-Cori* region to the terminus region (Arias-Cartin et al., 2017). Recently, another NAP “HupB” in *M. smegmatis* (Holowka et al., 2017) was shown to have a similar chromosome-wide gradient distribution. In the absence of GapR, both DNA replication and cell division are impaired (Arias-Cartin et al., 2017; Taylor et al., 2017). However, depletion of GapR only slightly affects global gene expression and most of the genes that are overexpressed belong to the DNA damage stress response and could be induced by indirect DNA damage. These observations argue that GapR is not primarily a transcription regulator. ChIP-seq analysis and fluorescence microscopy have shown that binding of GapR on the chromosome is dynamic and changes throughout the cell cycle. The strongest GapR peaks accumulate near *Cori* and downstream of *parB* near *parS* during the initiation of DNA replication (Taylor et al., 2017). Through this binding GapR somehow enhances the early slow phase of chromosome partitioning (Figure 1B), because without GapR, the *parS-Cori* region duplicates and then collapses into one focus before repeating the separation/partitioning process. This is the critical time when separation of the two chromosomes directs them to their alternative fates (Taylor et al., 2017). Subsequently, as the chromosome replicates and partitions, GapR localization correlates with the moving replisome and the replication fork seems to displace the protein from the DNA (Arias-Cartin et al., 2017). Consistent with these observations, X-ray protein crystallography has shown that two GapR dimers assemble to encircle DNA that must be overly twisted to fit inside the hole (Guo et al., 2018). Such overly twisted DNA is either found in front of the replication forks or downstream highly transcribed genes. Although the molecular details have still to be explored, it was proposed that once bound to the overly twisted DNA, GapR enhances or recruits the gyrase activity to dissipate (+) supercoiled DNA produced by replication forks and by RNA polymerase (Guo et al., 2018). Therefore, unlike most NAPs that primarily compact the nucleoid, GapR seems to primary facilitate nucleoid replication and partitioning perhaps at least in part by strategically directing DNA gyrase and perhaps other “molecular machines” including RNA and DNA polymerases.

## *C. Crescentus* Protein Popz is a Polar Organizing “Hub”

Multiple cell-cycle regulators act through the cell poles, and PopZ is their polar “hub protein” acting at the heart of chromosome replication and partitioning (Bowman et al., 2010). PopZ is an intrinsically disordered network protein that fills and forms special apical zones in the cytoplasm. Molecular recognition features “MoRFs” (Holmes et al., 2016) allow PopZ to engage and to localize many cell-cycle proteins. PopZ is initially found at the cell poles, where it binds ParB to anchor *parS* (Bowman et al., 2008; Ebersbach et al., 2008). In addition to this key function, PopZ serves as a platform for other cell-cycle regulators. For example, CtrA and its kinases CckA regulate chromosome replication. CtrA binds *Cori* and both CtrA and CckA are recruited to the stalked cell pole in a PopZ-dependent manner (Bowman et al., 2010; Holmes et al., 2016). Moreover, PopZ sequesters and restrains the CtrA-targeting protease ClpXP (Joshi et al., 2018). In the absence of PopZ, ClpXP exhibits unprecedently high CtrA degradation rates. Under normal conditions, the PopZ-recruited adaptor protein CdpR modulates ClpXP activity also by CckA-mediated phosphorylation. When PopZ is lost, CckA localization is hindered, and CdpR remains in its “active” dephosphorylated state. Consequently, overly active CdpR recruits more ClpXP to accelerate the proteolysis of CtrA. Interestingly, over-expression of PopZ also stimulates the proteolysis of CtrA but by a different mechanism. Under these abnormal conditions, CtrA and ClpXP are thought to concentrate at the cell pole and directly interact without using the CdpR adaptor (Joshi et al., 2018).

While CtrA inactivation is required for the initiation of chromosome replication in stalked cells (Figure 1A), its re-accumulation and phosphorylation in late S-phase are also required for cell-cycle transcription control and to prevent premature replication in the new swarmer cell compartment (Sanselicio et al., 2015). Accordingly, MopJ (motility PAS domain associated with DivJ) emerged as an important enhancing factor for CtrA accumulation (Sanselicio et al., 2015). At the cell poles, MopJ attenuates the DivJ-DivK-DivL kinase pathway that is also involved in the downregulation of CtrA through ClpXP. Once again, PopZ lies at the heart of this molecular interaction because the PopZ polar matrix localizes DivJ to the stalked pole, which in turn drives the polarization of DivK, DivL, and MopJ (Ebersbach et al., 2008).

During chromosome replication, the role of PopZ in partitioning switches from passive anchoring to an active participation in the movement of *parS-Cori*. Co-Immunoprecipitation experiments revealed that PopZ interacts directly with ParB, and a PopZ-ParB-*parS* complex presumably accounts for the initial polar anchoring/tethering at the early stalked pole (Bowman et al., 2008). Somehow the *parS-Cori* region is released from PopZ, and upon replication initiation, the duplicated DNA regions are separated such that one region seems to reattach, while the other moves slowly toward the quarter cell-length position. This corresponds to the slow phase of chromosome partitioning (Figure 1) that, as we described above, requires GapR but not ParA (Taylor et al., 2017). This is also the symmetry splitting point in the cell cycle that determines the subsequent fates of the chromosomes. Once this step is reached, the subsequent fast phase of partitioning uses ParA-ATPase activity. As the ParB-*parS* chromosome complex contacts DNA-bound ParA-ATP, the stimulated ATP hydrolysis causes subsequent ParA release. Such repeated interactions of binding and unbinding presumably cause the movement toward the new pole (Laloux and Jacobs-Wagner, 2013; Ptacin et al., 2014). Interestingly, the PopZ matrix directly sequesters the DNA-released ParA subunits at the new pole and then revives their ATP-bound state and their affinity for nucleoid DNA (Ptacin et al., 2014). This “recycling” or “rejuvenating” function of PopZ presumably enhances partitioning, since by concentrating and reactivating ParA-ATP dimers, PopZ will create a sharper ParA gradient that leads to the new cell pole. Interestingly, another cell pole “landmark” protein “TipN” shares functional redundancy with PopZ as it also recruits ParA to prevent reversal of the segregating ParB-*parS* complex (Ptacin et al., 2014). Accordingly, the ΔtipNΔpopZ double mutation is synthetically lethal (Schofield et al., 2010), and TipN polar localization is disrupted in the absence of PopZ (Ebersbach et al., 2008).

Further studies suggest an added layer of communication between ParA and PopZ. The redistribution of PopZ to the new swarmer pole (Figure 1) is coordinated with the arrival of the second ParB-*parS* focus at the new pole (Bowman et al., 2008; Ebersbach et al., 2008; Laloux and Jacobs-Wagner, 2013). Therefore, the ParA-dependent partitioning process somehow also drives the bi-polar organization of PopZ. In support of this notion, delayed partitioning caused by TipN depletion postponed PopZ accumulation at the new pole (Laloux and Jacobs-Wagner, 2013). ParA participates in the formation of the new PopZ matrix, as its loss disrupts PopZ bi-polarity. While other means of PopZ localization have been suggested such as self-organization by nucleoid occlusion (Ebersbach et al., 2008; Saberi and Emberly, 2010), these are clearly not enough, and a ParA-mediated PopZ-localization mechanism is required. If basal levels of ParA initiate PopZ recruitment, this may trigger a positive-feedback loop where ParA and PopZ will accumulate together through mutual support (Laloux and Jacobs-Wagner, 2013). As mentioned above, TipN also recruits ParA, and therefore, this polar landmark protein may also start or contribute to the growth of the PopZ matrix.

PopZ interactions are certainly complex yet robust, and however, this happens in wild-type *C. crescentus* cells, a new PopZ matrix always forms in time to meet and anchor the ParB-*parS* complex arriving at the new swarmer pole (Figure 1B). Interestingly, this cell-cycle pattern is very similar to that of the *V. cholerae* Chrom I, which is anchored through *parS1*-ParB1 to a polar PopZ-like protein called “HubP” (Yamaichi et al., 2012). Yet despite such a striking functional correspondence, HubP and PopZ are otherwise evolutionarily unrelated proteins.

The cell-cycle regulated zinc-finger protein ZitP offers yet another mechanism to control PopZ, independent of the *parABS* system (Berge et al., 2016). When ZitP is removed in a strain expressing a variant of PopZ that cannot bind ParB, bi-polar ParB fluorescent foci are rarely seen. However, the resupply of ZitP restores ParB foci at both cell poles, which implies the restoration of localized PopZ anchors (Berge et al., 2016). In this situation, the chromosome anchoring function may rely solely on ZitP since the PopZ variant is unable to bind ParB, but in wild-type cells, the role of ZitP in anchoring would be considered supportive. Normally, PopZ-bound ZitP indirectly binds to *parS*-flanking sites, where it functions to enhance ParB nucleation on the *parS* DNA. This assembly of ZitP-PopZ-ParB on the chromosome effectively restrains segregation (Berge et al., 2016).

It seems that the common theme for this multifaceted PopZ protein is its capacity for two-way interactions with many regulating and cell organizing proteins. For example, ZitP also relies on PopZ to recruit and position pilus biogenesis and swarming motility systems (Mignolet et al., 2016). In summary, PopZ is certainly a “hub” for cell-cycle communication that is yet to be fully explored as a mediator of crosstalk. Future studies promise new insights and new mechanisms of crosstalk between chromosome replication, partitioning, and probably the other landmarks of the cell cycle.

## Author Contributions

GM proposed, organized, and wrote the bulk of this review. KP wrote the section on GapR. PP wrote the section on PopZ.

### Conflict of Interest Statement

The authors declare that the research was conducted in the absence of any commercial or financial relationships that could be construed as a potential conflict of interest.
